# Cohort bias in predictive risk assessments of future criminal justice system involvement

**DOI:** 10.1073/pnas.2301990120

**Published:** 2023-05-30

**Authors:** Erika Montana, Daniel S. Nagin, Roland Neil, Robert J. Sampson

**Affiliations:** ^a^Heinz College, Carnegie Mellon University, Pittsburgh, PA 15213; ^b^Department of Criminology, University of Pennsylvania, Philadelphia, PA 19104; ^c^Department of Sociology, Harvard University, Cambridge, MA 02138

**Keywords:** risk assessment, criminal justice, bias, cohort, social change

## Abstract

Risk assessment instruments (RAIs) are widely used to aid high-stakes decision-making in criminal justice settings and other areas such as health care and child welfare. These tools, whether using machine learning or simpler algorithms, typically assume a time-invariant relationship between predictors and outcome. Because societies are themselves changing and not just individuals, this assumption may be violated in many behavioral settings, generating what we call cohort bias. Analyzing criminal histories in a cohort-sequential longitudinal study of children, we demonstrate that regardless of model type or predictor sets, a tool trained to predict the likelihood of arrest between the ages of 17 and 24 y on older birth cohorts systematically overpredicts the likelihood of arrest for younger birth cohorts over the period 1995 to 2020. Cohort bias is found for both relative and absolute risks, and it persists for all racial groups and within groups at highest risk for arrest. The results imply that cohort bias is an underappreciated mechanism generating inequality in contacts with the criminal legal system that is distinct from racial bias. Cohort bias is a challenge not only for predictive instruments with respect to crime and justice, but also for RAIs more broadly.

## Significance

Social science research and policy increasingly rely on predictive risk assessment instruments (RAIs), including those using machine-learning algorithms. This paper shows that the relationships between risk factors and future arrest are unstable over time when measured across sequential birth cohorts. As a result, prediction models that rely on risk factors are prone to systematic and substantial error. Such cohort bias, arising from the dynamics of social change, requires algorithmic updating and accounting for social factors affecting entire cohorts. Cohort bias can generate inequality in criminal justice contacts distinct from racial bias and has implications not only for the tailoring of RAIs but also for efforts aiming to provide preventative interventions to high-risk groups targeted based on individual-level risk factors alone.

Prediction is a central goal in the science of human behavior (1, 2). Risk assessment instruments (RAIs), whether based on simple scoring tools or sophisticated machine-learning algorithms, are widely used to aid high-stakes decision-making in diverse domains including health care (3), child welfare (4), and crime (5–9). RAIs are used in making life-altering treatment and disposition decisions in medical and judicial settings and may have similar life-altering impacts when used by social service agencies to determine whom to target for interventions. In predicting criminal behavior and legal system involvement, RAIs typically predict future risk using a combination of features measuring individual characteristics, family background, and prior criminal history (7–11).

In this paper, we argue that RAIs are challenged by a crucial but commonly neglected fact: As time goes on, it is not just individuals who age and develop, but societies themselves contemporaneously change. This fact implies that individuals’ future behaviors are not only a function of their stable traits, earlier-life circumstances, prior behaviors, and age, but also ongoing social change affecting all members of a birth cohort. That an algorithm’s performance can degrade over time is known, but implications of ongoing change are typically not recognized in real-world implementations of RAIs and more generally in the conceptualization of future risk. We therefore examine the power of social change to impact the performance of RAIs.

Processes of social change pose distinct challenges to the performance of RAIs because, regardless of how an RAI is built and used, the accuracy of its predictions relies on the fundamental assumption that the relationships between predictors and outcomes of interest are stable over time. Social change may undermine that stability in complex and unpredictable ways for people who grew up in different times, leading to what we term “cohort bias” in predictive risk assessments. Our analysis focuses on predicting official arrest; societal-wide change, however, is not particular to this outcome.

We test the implications of this argument with a study of the calibration and rank-ordering performance of models trained to predict arrest in early adulthood across multiple birth cohorts who came of age at different times over the period from 1995 to 2020. Arrest is both the first formal point of entry to criminal legal processing and a widely used measure of the risk of future criminal behavior and criminal legal involvement (12). Arrest prediction is thus a typical application of RAIs in criminal justice settings (11, 13, 14). Such predictions exemplify a high-stakes application of RAIs in that false negatives may allow damaging crimes to occur, and false positives may result in harmful and unfair targeting of individuals for crimes they were never going to commit. We demonstrate that cohort bias is present in both relative and absolute levels of risk. Cohort bias in risk level is particularly important because the social costs of predictive errors depend on biases in risk level.

## Research Questions about Cohort Bias

Much research on crime has centered on identifying early-life, individual-level, psychosocial, and neighborhood predictors of criminal involvement in adolescence and beyond. Such predictors include personal circumstances such as family instability and poverty, psychological characteristics such as low self-control, and growing up in poor neighborhoods (15). While the association between many such features with later-life criminal involvement is well established, whether the predictive strength of these features is constant across historical periods is uncertain. This is an important gap given that societal-level crime rates and broader social conditions are highly variable, as evidenced by the sustained drop in crime beginning in the early 1990s in the United States and other countries across the world (16, 17).

Researchers using data from a multi-cohort study, the Project on Human Development in Chicago Neighborhoods (PHDCN), have demonstrated large differences in age-specific arrest frequency and prevalence between birth cohorts separated by as little as 10 y (18, 19). They also show that well-established predictors of crime, such as growing up in poverty, self-control, sex, and race/ethnicity, cannot explain these cohort differences in arrest patterns. The cohort effects they identify are as large or in most cases larger than these individual-level risk factors in predicting arrest prevalence in young adulthood. The results suggest that the dynamics of social change are an important but neglected factor in the explanation and prediction of future involvement in crime.

Our research builds upon and extends these analyses by examining whether an RAI trained on individual-level features of an older age cohort accurately predicts the likelihood of arrest of a younger cohort. We define systematic differences between the actual arrest patterns of the younger cohort compared to their predicted arrest patterns based on RAIs trained on the data of an older cohort as cohort bias.

We address three questions. First, is there evidence of cohort bias? We test for this bias in both relative and absolute risk predictions. Not only do we find cohort bias, but it is substantial. The RAIs trained on the older cohort, regardless of model type and feature set specification that include variables measured from early life to late adolescence, overpredict the probability of arrest of the younger cohort by as much as 89%. Second, how is this cohort bias related to racial bias, a more commonly studied form of bias in machine-learning research (8, 9, 20)? We find substantial cohort bias within all the three racial–ethnic groups studied—White and others, Latinos, and Blacks—which establishes that cohort bias is distinct from racial biases. Third, we ask whether cohort bias can be mitigated by targeting high-risk groups either by accounting for arrest in adolescence or limiting predictions to those with low levels of self-control. We find that cohort bias persists even when measures of arrest from immediately before the ages for which we predict arrest are included as predictors and even when limiting analysis to high-risk participants.

Our aim is to help improve the science and use of RAIs in justice and other high-stakes contexts regarding human behavior. To identify possible mitigation strategies of cohort bias arising from social change in RAIs, we investigate whether the statistical relationship between predictors and arrest prevalence is stable across cohorts. We find that it is not, which implies that correcting for cohort bias requires more than an intercept adjustment to account for the overall trend in crime rates (21).

In high-stakes decision contexts, RAIs have the potential to do harm if not well calibrated. While human judgement is also fraught with biases, an RAI can make millions more judgments in its “lifetime,” amplifying its impact. Though our analysis uses population data with a criminal legal outcome, it is likely that the effects of social change are far reaching, implying that cohort bias may be found in RAIs used to predict recidivism among offending populations as well as those used to target individuals for early interventions.

## Materials and Methods

The data used come from an extension of the PHDCN and include 1,057 individuals from four different age cohorts (22). The study began in the mid-1990s with a representative sample of children, ranging from newborn to 18 y old, drawn from a representative sample of Chicago neighborhoods. Detailed in-home assessments were conducted to collect data on these children, their families, and their neighborhoods. Two further waves of data collection were conducted over approximately 2.5-y intervals.

Our work uses data from four age cohorts that were randomly sampled from wave three participants and reinterviewed for a fourth wave between 2011 and 2013, and on whom criminal records were later collected through 2020. These participants were 0, 9, 12, and 15 y old when the study began. We refer to the infant or age “0” cohort as the younger cohort; participants in the younger cohort were born in the mid-1990s. The 9-, 12-, and 15-y-old age cohorts are combined into the older cohort, which contains individuals born between 1979 and 1988, creating a 6- to 17-y age difference between individuals in the younger cohort and any individual in the older cohort. The fourth wave interview collected detailed information on a wide array of topics including information about behavior on the, by then adolescent, younger cohort. Adult respondents provided written or verbal consent to interviews. The Harvard University institutional review board approved data collection and analyses. Arrest records were collected from the Criminal History Record Information in Illinois that covered the period 1995 through 2020. They were matched to wave four participants by name and date of birth. The study followed Department of Justice human subject protection regulation 28 CFR Part 46.

The combined dataset includes rich features related to participants’ personal, early-life family, and neighborhood characteristics as well as arrest data for all participants, in all cohorts, between the ages of 17 and 24 y. The primary dependent variable is a binary variable indicating whether the individual was arrested as a young adult, between the ages of 17 and 24 y for feature sets without arrest history indicators, and between the ages of 19 and 24 y for feature sets with arrest history indicators. While arrests are not a direct measure of criminal involvement, they are commonly used in criminological research and are frequently the target of prediction applications in criminal justice settings (11).

Independent variables, or features in the machine-learning nomenclature, include sociodemographic information about the participant (sex, race/ethnicity, and caretaker’s immigrant generation), psychosocial characteristics (anxiety/depression, aggression, and low self-control), family characteristics (such as family size, household income, and parental education level), and neighborhood characteristics (such as poverty rates, college education rates, and violent crime rates). *SI Appendix*, Table S1 provides further information on the measures as well as respondents’ ages when the data were collected. Except for the time-invariant features, all features were measured at approximately the same ages across cohorts. The features span a period from childhood to just prior to the initial age of the outcome variable being predicted, arrest between 17 and 24. Furthermore, the dataset includes many of the classic childhood, family, and neighborhood predictors of crime (15, 23, 24). There were initially four categories of race and ethnicity: White, Black, Latino, and Other. Because there were few “Other” observations (n = 44), that category was combined with White. Descriptive data on the main feature set by cohort are shown in *SI Appendix*, Table S2.

Missing data are infrequent and there are no missing values in the dependent variable. Over 58% of the participants have no missing values, and an additional 12% are missing information on only one item. Only 78 participants (7%) have substantial missing information, in that they are missing values for more than 20% of the items. The variable with the highest level of missingness is household income; it is missing 142 values, 13% of the sample. No other variable is missing for more than 10% of the participants. Missing data were imputed using a k-nearest neighbor algorithm with k = 5.

### Model Estimation.

To identify the impact of different sets of covariates on observed cohort bias, two feature sets were created. We call one of these sets the classic risk-factor feature set, which is classic in the sense that it includes features that prior literature has identified as strongly correlated with crime and legal system involvement (23–25). These features measure characteristics of the individuals themselves and their immediate family and community environment. The classic risk-factor feature set includes sex, race, adolescent self-control, family poverty (as indicated by the receipt of Temporary Assistance for Needy Families, or TANF), caregiver marital status, and caregiver immigrant generation. The second feature set, which we call the full set, consists of thirty-five features including the classic risk-factor set plus other established, early-life indicators of future criminal involvement, individual psychosocial characteristics, as well as childhood community-level characteristics. Measures such as these are often used alongside criminal record histories as predictors for real-world RAIs (10).

Binary classification models were trained on the older cohort to predict whether respondents were arrested between the ages of 17 and 24 y. Models evaluated include logistic regression, lasso and ridge regularized logistic regressions, and random forests (26). Lasso and ridge regression are methods of shrinking coefficients associated with collinear predictors. These methods are designed to improve predictive performance for new cases by preventing models from overfitting, that is, fitting noise in the training sample. Random forests are a form of ensemble decision tree algorithm that allows for highly nonlinear and interactive predictions with low likelihood of overfitting.

Model performance on both younger and older cohorts was evaluated in several ways. Analyses rely primarily on calibration plots based on linear regressions to evaluate shifts in predictive accuracy for models trained on the older cohort when performance is evaluated on the younger as opposed to the older cohorts.

## Results

### Assessing the Degree of Cohort Bias.

Regardless of the method used or features included in the model, cohort bias is consistently found when predicting arrest from ages 17 to 24 y and also as described below other age ranges. For parsimony, we report results for logistic regression using the classic risk-factor feature set and the lasso logistic regression applied to the full set of predictive features. Sensitivity tests, including random forest and ridge regression models, yield similar results (*SI Appendix*, Fig. S1).

**Fig. 1.**
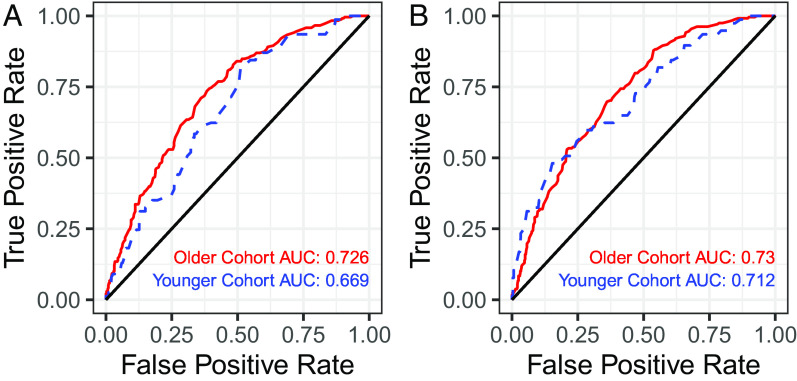
ROC curves showing performance of logistic regression models. Models were trained on the older cohort. Performance on the older cohort is shown in red, while performance on the younger cohort is shown in blue. Results are shown for both (*A*) an unregularized logistic regression using the classic risk-factor feature set and (*B*) a lasso logistic regression using the full feature set.

Fig. 1 reports receiver operating characteristic (ROC) curves for the classic risk-factor logistic regression model (Fig. 1*A*) and the full lasso logistic regression model (Fig. 1*B*). Each panel reports two ROC curves, one for the model trained on the older cohort applied to the older cohort and another for that same model applied to the younger cohort. Based on area under the curve (AUC) values, our models perform as well or better than those used in practice (13, 27). The classic risk-factor regression in the top panel has an AUC of 0.726 when applied to the older cohort, while the full model has an only slightly higher AUC of 0.730. These same models have lower AUC values when applied to the younger cohort, 0.669 and 0.712, respectively. These results suggest that little predictive capacity is gained by adding features beyond those already included in the classic risk-factor model.

**Fig. 2.**
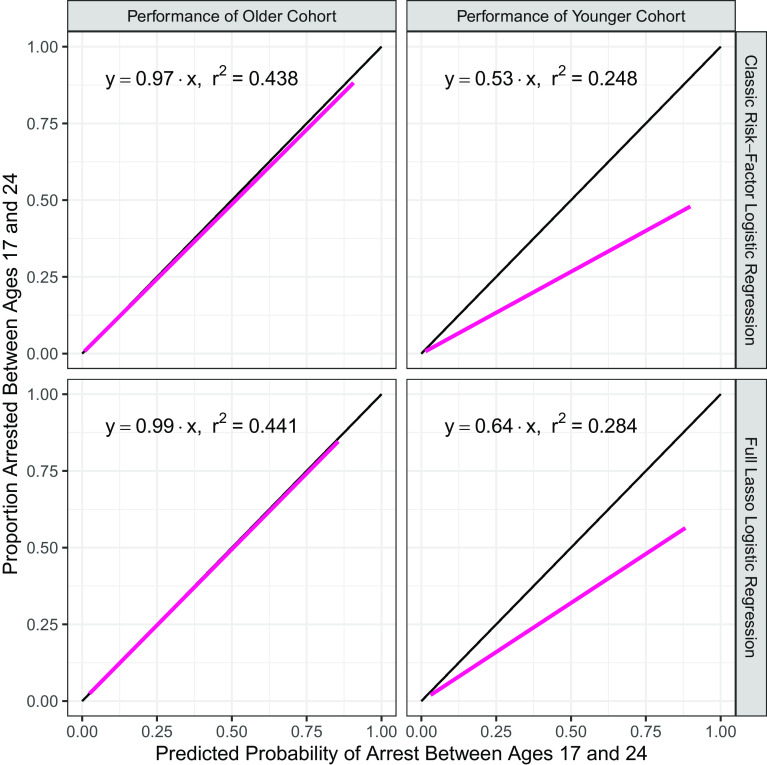
Calibration plots for models trained on older cohort. Calibration plots for the classic risk-factor unregularized logistic regression model (*Top*) and the full lasso logistic regression model (*Bottom*). Models were trained on the older cohort, and performance on the older cohort is shown on the *Left*, while performance on the younger cohort is shown on the *Right*. The regression line falls below the ideal 45° line indicating persistent overprediction of the likelihood of arrest for the younger cohort in both models.

Our purpose, however, is not to compare the predictive capabilities of alternative prediction methods but to test for cohort bias. Fig. 2 addresses this question with calibration plots that compare predictions of the probability of arrest from ages 17 to 24 y with the proportion arrested at those ages. Each plot is constructed by regressing the actual outcome of arrest between ages 17 and 24 y on the predicted probability for a slope-only regression. For a perfectly calibrated model, this line would fall on the black 45° line and have a slope of 1. The first row in Fig. 2 shows the calibration results of the classic risk-factor logistic regression, and the second row shows the results from the full lasso logistic regression. The first column shows the calibration of a model trained on the older cohort for the older cohort, and the second column shows the calibration of the same model when used to generate predictions for the younger cohort.

Fig. 2 shows that the models trained on the older cohort are well calibrated to that cohort with slopes of 0.97 and 0.99 for the classic risk-factor logistic regression and the full lasso logistic regression, respectively. Being well calibrated means that on average the predicted probability of arrest from the model is the same as the proportion of participants who actually are arrested at that probability level.

However, when these same models are applied to the younger cohort, both models systematically overpredict the probability of arrest. For the logistic and lasso logistic regression models, the slope estimates are 0.53 and 0.64, respectively, which implies that older cohort trained models are overpredicting arrest probability by about 89% for the logistic regression model and 56% for the lasso logistic regression model. Results for the ridge logistic and random forest models are similar (*SI Appendix*, Fig. S1). Thus, regardless of algorithm or feature set used, there is consistently evidence of cohort bias. While cohort bias could produce either underprediction or overprediction, in this application, it results in a substantial overprediction of the probability of arrest. For reasons elaborated upon in the *Discussion* section, in criminal justice applications, the costs of such overpredictions of risk level are high.

We also examined whether cohort bias contaminates predictions of relative risk rankings. To do so, we compared risk rankings of younger cohort members resulting from application of an RAI trained on the older cohort with rankings of an RAI trained on the younger cohort themselves based on a 10-fold cross validation design. This analysis was conducted for each of the four estimation methods on both the classic risk-factor feature set and the full feature set.

**Fig. 3.**
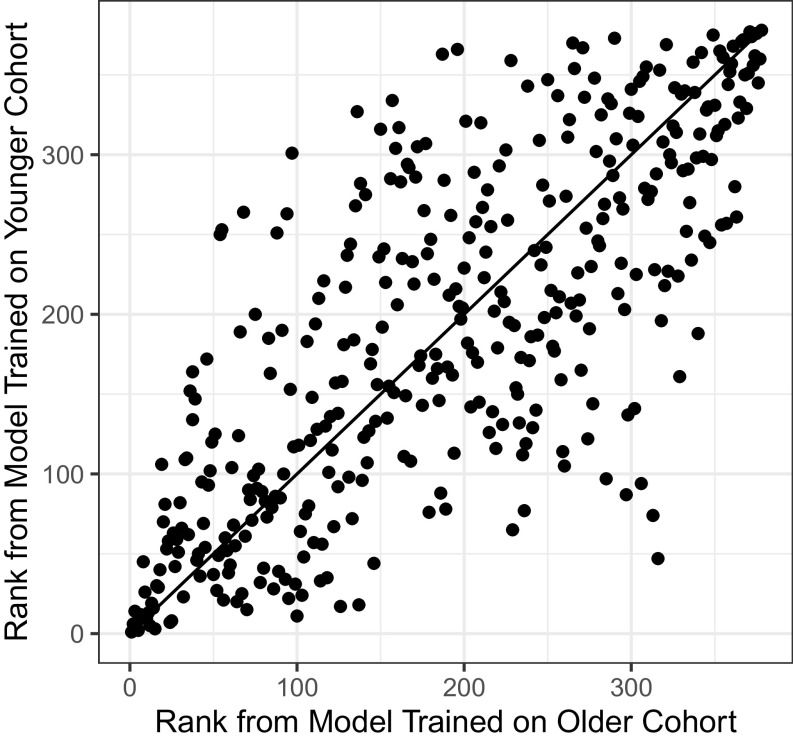
Comparison of rankings between full lasso logistic regression models trained on different cohorts. Plot of younger cohort, risk rankings produced by the full lasso logistic regression model trained on the older cohort (*x* axis) and the same model trained on the younger cohort (*y* axis). Both the plot and the Spearman’s correlation value of 0.76 show that while the two sets of rankings are correlated, there are substantial ranking differences between the model produced by the older cohort and that produced by the younger cohort.

Across the resulting eight separate analyses, the average overlap of membership in the highest quartile of risk was only 53%, with a low of 44% for the logistic regression analysis conducted on the full feature set, to a high of 64% for the lasso logistic regression on the full feature set. Fig. 3 is a scatter plot of the predictions for this best performing model. While there is a clear positive association, the variability about the 45° line is large (rank-order correlation = 0.76; correlations across the eight analyses range from 0.46 to 0.76). We also compared the risk rankings of the older cohort members using the four different methods and two different feature sets. This analysis produced overlaps in the top risk quartile that were much higher than those of the across-cohort analysis, ranging from 65 to 98%, with corresponding rank-order correlations between 0.77 and 0.99. This suggests that the decline in rank-ordering performance is due to cohort bias, rather than an inherent sensitivity of rank orderings across model specifications.

### Assessing Whether Cohort Bias Is Distinct from Racial Bias.

**Fig. 4.**
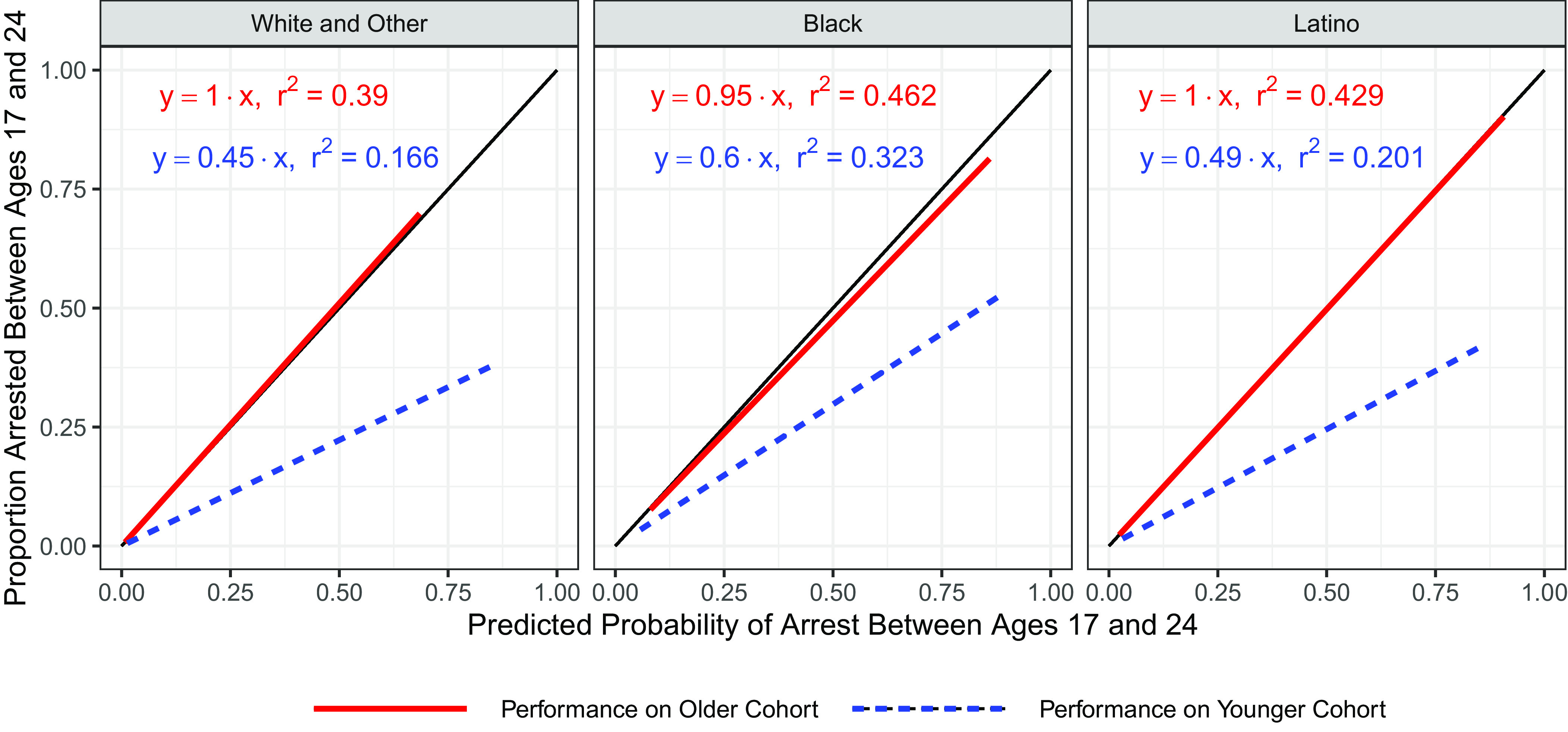
Calibration plots for classic risk-factor logistic regression model trained on older cohort by race. Performance on the older cohort of the classic risk-factor logistic regression model trained on the older cohort is shown in red, and performance on the younger cohort is shown in blue. Systematic overprediction of the likelihood of arrest is observed across racial groups.

To test whether cohort bias is a separate source of bias from racial bias, we applied the same analyses as in the preceding section to three nonoverlapping racial/ethnic groups in the PHDCN data: Whites and others (mostly Asians) (n = 242), Blacks (n = 386), and Latinos (n = 424). The results for the classic risk-factor logistic regression model are reported in Fig. 4, and the full lasso logistic regression results are in *SI Appendix*, Fig. S2. Across all racial groups, the models trained on the older cohort are well calibrated to that cohort with slope estimates of 1, 0.95, and 1 for the logistic regression and 0.95, 0.97, and 1.1 for the lasso logistic regression model. However, both models systematically overpredict the likelihood of arrest across racial groups with slope estimates of 0.45, 0.60, and 0.49 for the logistic regression and 0.56, 0.7, and 0.62 for the lasso logistic regression model. While the magnitude of cohort bias varies somewhat between racial groups, its persistence across groups indicates that the observed cohort bias cannot be a manifestation of racial biases in the data or algorithms. Instead, cohort bias is a distinct form of algorithmic bias.

### Assessing Cohort Bias within High-Risk Groups.

It is possible that the cohort bias that we observe is the product of a large decline in arrest prevalence for low-risk groups without substantial decline in arrest prevalence for high-risk groups. If so, cohort bias could be mitigated by training the RAI using data for the high-risk group or adding a feature which identifies membership in the high-risk group. Here, we analyze the calibration shift between cohorts for three different definitions of high-risk groups. The first method involves adding a feature to indicate prior arrest, which allows the model to adjust predictions for higher-risk participants. The second method focuses on individuals who score more than two SDs above the mean on tests of low self-control, and the third method focuses on individuals in the top quartile of arrest probability based on a model trained on the older cohort.

**Fig. 5.**
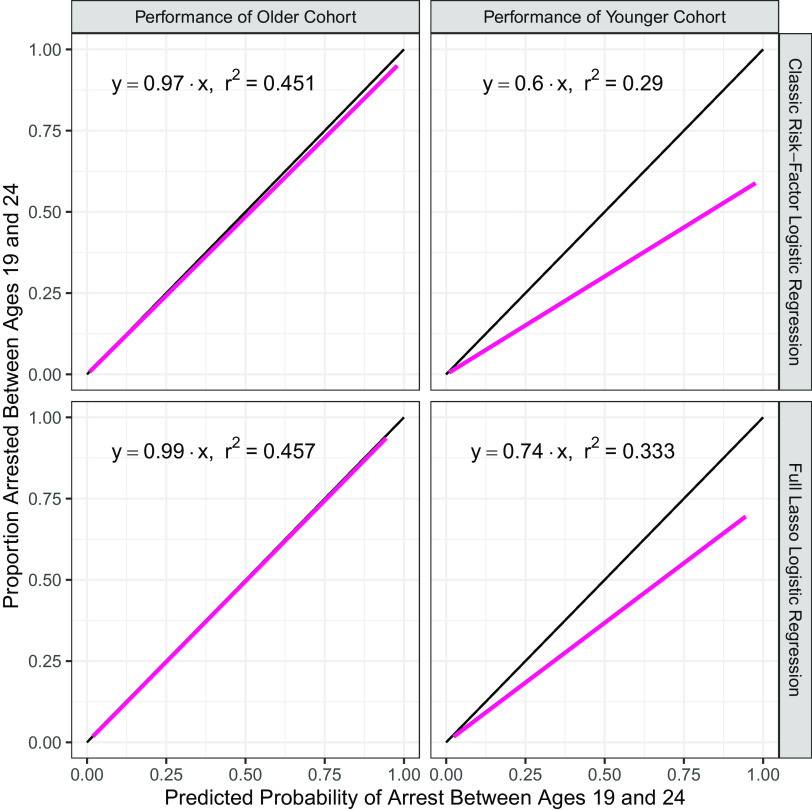
Calibration plots for models including an adolescent arrest history predictor and trained on older cohort. Calibration plots for two models which include a binary variable indicating arrest at age 17 or 18 y: the classic risk-factor logistic regression model (*Top*) and the full lasso regularized logistic regression model (*Bottom*). The left column shows the performance of these models on the older cohort on which they were trained. The right column shows the performance of the same models on the younger cohort. When arrest history is included as a predictor, models still overpredict arrest likelihood for the younger cohort.

To test whether cohort bias persisted for individuals with prior arrests, the feature set was expanded to include a binary indicator of arrest between ages 17 and 18 y in models predicting arrest between ages 19 and 24 y. Fig. 5 shows the calibration plots from these regressions. While the bias is mitigated by including prior arrest—a strong predictor of future arrest—cohort bias is still evident, as is evidenced by the slopes of the classic risk-factor (0.6) and lasso models (0.74), which remain well below 1.

In practice, many RAIs have a smaller prediction window than those trained as part of this analysis (i.e., they predict fewer than 7 y ahead) and often rely heavily on criminal history. To investigate the sensitivity of cohort bias to alternative specifications of arrest history and the dependent variable, two additional specifications were examined. The first shortened the prediction window to arrest between ages 22 and 24 y and lengthened the arrest history used as a predictor to include the ages from 17 to 21 y. The second formulation included juvenile arrest history from the ages of 10 to 16 y as a predictor of arrest between the ages of 17 and 24 y. Models for this specification could only be trained on a subset of the older cohort (the age 9 cohort) due to incomplete juvenile records for some of the oldest members of the PHDCN sample. The results of these two formulations mirror those shown in Fig. 5 and are available in *SI Appendix*, Figs. S3 and S4, respectively.

**Fig. 6.**
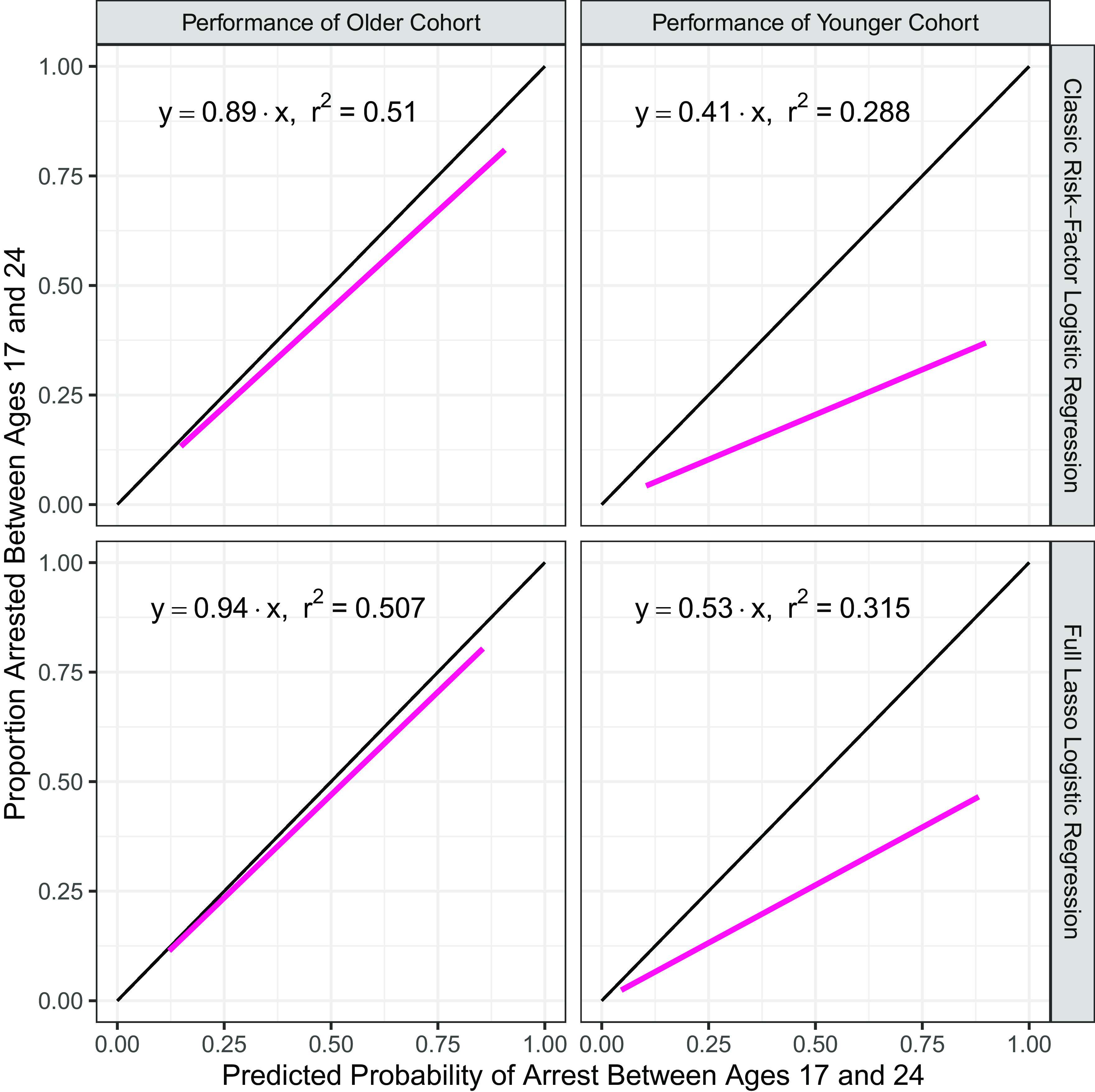
Calibration plots for models trained on older cohort applied to individuals who are two SDs below the average on self-control. The *Top* row shows the calibration of the classic risk-factor logistic regression model with performance for the older cohort on the *Left* and performance for the younger cohort on the *Right*. The *Bottom* row shows the performance of full lasso logistic regression model. The deviation of the regression line from the diagonal in the right column indicates that when considering only individuals with low self-control, cohort bias is observed.

Fig. 6 reports the calibration plots for high-risk groups defined by individuals who score more than two SDs above the mean for low self-control. As before, cohort bias is evident. Similar results are shown in *SI Appendix*, Fig. S5, which reports calibration plots based on individuals with a high predicted probability of arrest (top quartile) from model covariates. Hence, regardless of how high-risk individuals are defined, models trained on the older cohort systematically overpredict the likelihood of future arrest for the younger cohort.

### Assessing Predictive Stability of Risk Factors.

One explanation for the observed bias is that across cohorts the prediction model’s intercept is changing over time, but the parameters measuring the predictive impact of features remain unchanged. If this were the case, cohort bias could be corrected in some fashion by accounting for the intercept drift. Alternatively, feature parameters themselves may be changing across cohort due to changes in the underlying covariance structure between features and the outcome, which would greatly complicate adapting the model.

To identify the source of cohort bias, we compared logistic regression models trained on the older cohort to those trained on the younger cohort. Table 1 reports the coefficients, SEs, and *P*-values for each of these models. While there is a significant shift in the intercept value, there are also statistically significant changes in other coefficients. Coefficients for caregiver marital status, poverty, and immigrant generation, for example, change values between models trained on the older and younger cohorts. These shifts indicate that the underlying covariance relating predictors to arrest probability is changing over time.

**Table 1.** Coefficient shift between logistic regression model trained on older cohort and logistic regression model trained on the younger cohort

	Model trained on older cohort			Model trained on younger cohort			Coefficient difference
	Coefficient estimate	SE	*P*-value	Coefficient estimate	SE	*P*-value	*P*-value
Intercept	−0.770	0.094	0.000***	−1.696	0.169	0.000***	0.000***
Sex: Male	0.651	0.092	0.000***	0.615	0.152	0.000***	0.841
Race/ethnicity: Black	0.210	0.133	0.113	0.315	0.197	0.109	0.658
Race/ethnicity: Latino	0.427	0.158	0.007***	−0.061	0.224	0.787	0.075*
Caregiver immigrant generation: 2nd	−0.679	0.149	0.000***	−0.329	0.234	0.16	0.206
Caregiver immigrant generation: 1st	−0.498	0.153	0.001***	0.453	0.231	0.049**	0.001***
Family poverty	0.209	0.092	0.023**	−0.112	0.154	0.467	0.074*
Caregiver marital status: DSW	−0.097	0.100	0.333	0.453	0.149	0.002***	0.002***
Caregiver marital status: Cohabitating	0.184	0.089	0.038**	0.164	0.117	0.159	0.89
Caregiver marital status: Single	0.162	0.100	0.105	0.675	0.160	0.000***	0.007***
Low self-control	0.300	0.090	0.001***	0.325	0.143	0.023**	0.881

Under alternative specifications, such as a reduced model or the full risk set, the specific coefficients that shift can vary. The key finding is that the overall covariance structure is unstable over time.

^*^P < 0.1,

^**^P < 0.05,

^***^P < 0.01.

Theoretically, social change could be captured by including features connected to the time and place of the child’s development. However, we did not find that adding additional neighborhood census variables (poverty rate, percent Black, percent Hispanic, owner-occupied rate, and percent of homes with children) collected at distinct ages (9, 13, and 17 y) as predictors had any mitigating effect on the observed cohort bias (*SI Appendix*, Fig. S6). Another possibility is that cohort bias is the result of shifting law enforcement practices, particularly in highly discretionary drug arrest patterns. While this is a potential source of cohort bias in RAIs, repeating analyses but excluding drug arrests yields nearly identical results to analyses that include drug arrests (*SI Appendix*, Fig. S7).

## Discussion

Societies, just like the individuals who compose them, change over time. Although seemingly obvious, social change poses an important and underappreciated challenge for predictive RAIs. The accuracy of their predictions depends on the assumption that the relationship between the outcome of interest and predictors is stable over time. Our analysis has shown that in predicting arrest between the ages of 17 and 24 y as well as other age ranges for a representative population sample, this assumption is consistently violated for a variety of model types and feature sets. The result is cohort bias between cohorts separated by as little as 10 y or less, producing substantial overestimation of the probability of arrest for younger cohorts.

We found that cohort bias exists for all racial groups. Cohort bias is thus a source of inequality separate from mechanisms related to racial or ethnic bias and must be separately considered and addressed. In circumstances in which cohort bias generates overpredictions of the risk of criminal activity, individuals may be denied bail, sentenced more severely, or be denied parole due to an inflated prediction of crime risk (28), potentially triggering further involvement with the criminal justice system and in turn exacerbating racial inequalities.

While cohort bias might be attributable to unobserved individual- or neighborhood-level features, we judge this explanation unlikely not only because of the magnitude of the bias but also because the models we fit used an extensive set of predictors, including many of the most well-established risk factors for arrest. Furthermore, even if such unobserved characteristics are the root cause of the observed model degradation, knowing this would not solve the problem for real-world RAIs used today as they are constructed from much more limited feature sets and generally rely on administrative data (9, 10). Cohort bias when predicting arrests could also be driven by changing patterns of criminal behavior or police enforcement. A prior analysis of the data used in this study found that both were important in roughly equal proportion in producing cohort differences in arrests (18).

Our analysis is based on a population sample. The cohort differences in arrests in this sample reflect a widespread decline in arrests that took place in Chicago from the 1990s until recently, with major arrest types falling by at least two-thirds; similar declines happened at least through the 1990s for most of America (16, 18, 29). Many of these trends have since flatlined or reversed, underscoring how the nature of cohort bias cannot be known a priori given social changes, which can be abrupt and countervailing. An important next step is to perform similar analyses on criminal justice–involved samples based on arrest or conviction records to determine whether similar cohort biases exist in those contexts. More broadly, we expect that this work has implications for other public policy fields such as research on addiction and teen pregnancies where similarly large cohort rate differences are well documented (30, 31).

Even though in the criminal justice setting RAIs typically predict relative risk tiers, the costs of misclassification are a function of absolute risk level, a situation that holds for other areas too, such as health. For example, RAIs routinely used in high-stakes medical decision-making such as the Acute Physiology and Chronic Health Evaluation (APACHE) mortality score (32) and SOFA organ failure assessment score (33) predict mortality probability, not relative rankings of risk. This is because medical decisions such as the withdrawal of life support are made based on predictions of the patient’s probability of survival not on the patient’s probability of survival relative to other patients. In the context of arrest, an algorithm that rank ordered properly without calibration might successfully pick out the highest risk people in a group even if those people did not actually pose a high risk. While in the current case we found cohort bias in both relative and absolute rankings, these examples highlight why it is so important to examine models’ performances in terms of absolute risk levels, rather than just relative ranking.

Our findings also have implications for benefit–cost analyses of interventions intended to avert offending. Benefit–cost analyses of treatment interventions assign monetary values to the difference in outcome between the treated and control groups, not its relative frequency between the treatment and control groups. A standard example is the benefit–cost analysis of the Perry Pre-School Program by (34) which concluded that participants in a preschool program designed to improve cognitive functioning had significantly lower rates of adult criminal involvement compared to nonparticipants. The treatment and control groups were born circa 1960 at the outset of sustained crime increases in the United States. Crime reduction and other benefits were estimated to exceed program costs by a factor of 7. Whether that ratio would hold for children born into a period of declining crime rates is uncertain. The changing prevalence across birth cohorts for criminal involvement implies that the benefit–costs based on an earlier birth cohort may not apply to future birth cohorts.

Our purpose is not to question the value of RAIs as aids for decision-making. After all, the alternative to RAIs is decision-making by humans (such as judges) who may also suffer from cohort bias. We see three complementary approaches for mitigating, if not remedying, cohort bias to ensure that RAI tools are as effective and fair as possible.

The first is to find and construct measures of the social forces which may influence the behavior of entire cohorts and include those measures as model features. Identification of such measures is important not only for improving the predictive performance of RAIs but also to advance knowledge about the contextual influences of human behavior. Whether humans update their beliefs to account for contextual social change is a related topic for future work. For example, how do humans cognitively recognize and conceptualize cohort bias? In the present case, how do police, prosecutors, judges, and risk classifiers in general develop mental models of how the world works based on their experiences and observations, and how good are they at recognizing that the world is changing over time and consequently updating their beliefs?

A second approach involves adapting the prediction instrument to account for changes in the covariance structure relating potential features to outcomes. Such a method could involve reweighting training data based on the age of each observation to create an exponential smoothing effect. The precise adaptation method would need to be tested and validated.

Third, the most straightforward approach to mitigating cohort bias is to ensure that RAIs are updated frequently. While further research is needed to determine what update frequency is necessary, it is common for RAIs to go ten or even more than 15 y between updates. For example, the instrument used by the New York City Criminal Justice Agency to predict pretrial risk of nonappearance was developed in 2003 and used without updating until 2020 (35).

In sum, substantial social science research centers on the identification of predictive risk factors. Our analysis suggests that the relationships between identified risk factors and problem behaviors are not stable over time. By implication, the performance of prediction models that rely on these risk factors is also unlikely to be stable over time. This dynamic relationship between risk factors and problem behaviors has important implications not only for the tailoring of RAIs but also for a large body of prevention research that aims to provide interventions to high-risk groups targeted based on individual-level risk factors. In a well-known case, changes to Google’s search algorithm led Google Flu Trends to severely overpredict flu patterns over time (36). A key lesson was that predictive models can fail when changes to the relationship between predictors and outcomes are not considered. We have shown that RAIs are likely to similarly fail over time, for more recent cohorts, if the dynamics of social change are ignored.

## Supplementary Material

Appendix 01 (PDF)

## Data Availability

Restricted-access data and replication code are available in Harvard Dataverse (https://doi.org/10.7910/DVN/BGXN3B) (37).
