# Supplementary material for: Cohort bias in predictive risk assessments of future criminal justice system involvement
Source: Proc Natl Acad Sci U S A. 2023 May 30;120(23):e2301990120. doi: 10.1073/pnas.2301990120 (PMC10265989; doi:10.1073/pnas.2301990120)
Supplement: Supplementary file 1 — Appendix 01 (PDF) [file pnas.2301990120.sapp.pdf]

PNAS
www.pnas.org

Supporting Information (SI) Appendix:

Cohort Bias in Predictive Risk Assessments of Future Criminal Justice System Involvement

Erika Montana[a], Daniel S. Nagin[a], Roland Neil[b], and Robert J. Sampson[c,1]

[a]Carnegie Mellon University, Heinz College, Pittsburgh, PA 15213

[b]University of Pennsylvania, Department of Criminology, Philadelphia, PA 19104

[c]Harvard University, Department of Sociology, Cambridge, MA 02138

[1]To whom correspondence may be addressed. Email: rsampson@wjh.harvard.edu.

This PDF file includes:

Figures S1 to S7

Tables S1 to S2

Table S1. Features included in predictive models. The second column lists the features available with levels for categorical variables listed in parentheses. The third column indicates which features were used in the classic risk-factor models. All features listed are included in the full models. The fourth column indicates the average ages when these features were measured. Measures of predictive features span from early childhood through late adolescence. Because our quantities of interest are predictions of arrest among the cohort study members, and to be consistent with how risk assessment tools are typically used, features and analyses of these measures are based on unweighted data. Results using sampling design and attrition weights nonetheless converge and yield similar conclusions.

Variable Type	Variables and Descriptions	Predictor for Classic Risk-Factor Model	Age of Measurement
Time Invariant Demographics	Race/Ethnicity ("White or Other", "Black", and "Latino")	Yes	Measured at age 0 for younger cohort and 9-, 12-, and 15-years old for the older cohort members
	Sex (Male, Female)	Yes	
	Caregiver Immigrant Generation (1st, 2nd, 3rd generation)	Yes	
	Caregiver Age at Subject Birth (Less than 23 years, 23-30 years, More than 30 years)	No	
Parent and Family Characteristics	Parental Employment Status (Employed, Unemployed)	No	15-17 years old
	Family Poverty (TANF recipient, Not TANF recipient)	Yes	
	Parental Education (less than high school, high school diploma, some college, college degree or above)	No	
	Household Income (lower quartile, middle class, upper quartile)	No	
	Parental Marital Status (Married, Cohabitating, Single, Divorced/Widowed/Separated)	Yes	
	Family Residential Stability (years at current address) (Less than 2 years, 2-5 years, More than 5 years)	No	
	Household Size (2-3 people, 4 people, 5-6 people, 7 or more people)	No	
	Homeownership (Owned their home, Did not own their home)	No	
Adolescent Behavioral Disposition	Anxiety/Depression	No	15-17 years old
	Low Self-control/Impulsivity	Yes	
	Aggression/Antisocial Behavior	No	
Neighborhood Crime and Punishment	Homicide Rate	No	6-10 years old
	Robbery Rate	No	
	Weapon Offense Rate	No	
	Incarceration Rate	No	
Neighborhood Census Tract Data	Percent Black	No	Remeasured at 9, 13, and 17 years old
	Percent Hispanic	No	
	Concentrated Poverty (percent below the poverty line)	No	
	Percent of Homes with Children Under 18	No	
	Owner-Occupied Rate	No	
Criminal Justice System Contact	Arrest History	Yes	Measured between 17 and 24 years old for each subject

Table S2. Descriptive statistics on classic risk factors by cohort.

	**Older Cohort**	**Younger Cohort**	**P-Value**
	n = 679	n = 378	
Race/Ethnicity			0.167
White and Other	21.4%	26.5%	
Black	37.6%	34.7%	
Latino	41.1%	38.9%	
NA	0%	0%	
Sex: Male	47.9%	50.5%	0.443
Caregiver Immigrant Generation			0.301
3rd Generation	52.9%	55%	
2nd Generation	8.5%	10.3%	
1st Generation	37.6%	33.1%	
NA	1%	1.6%	
Family Poverty	0.1 (0.3)	0.1 (0.3)	0.453
Caregiver Marital Status			0.01
Married	56.1%	46%	
Cohabitating	5%	9.3%	
Divorced/Separated/Widowed	18.9%	19.8%	
Single	17.1%	14.8%	
NA	2.9%	10.1%	
Low Self Control/Impulsivity	-0.0 (1.0)	0.1 (1.0)	0.228
Arrested between ages of 17 and 24: Arrested	35.1%	20.4%	<.001

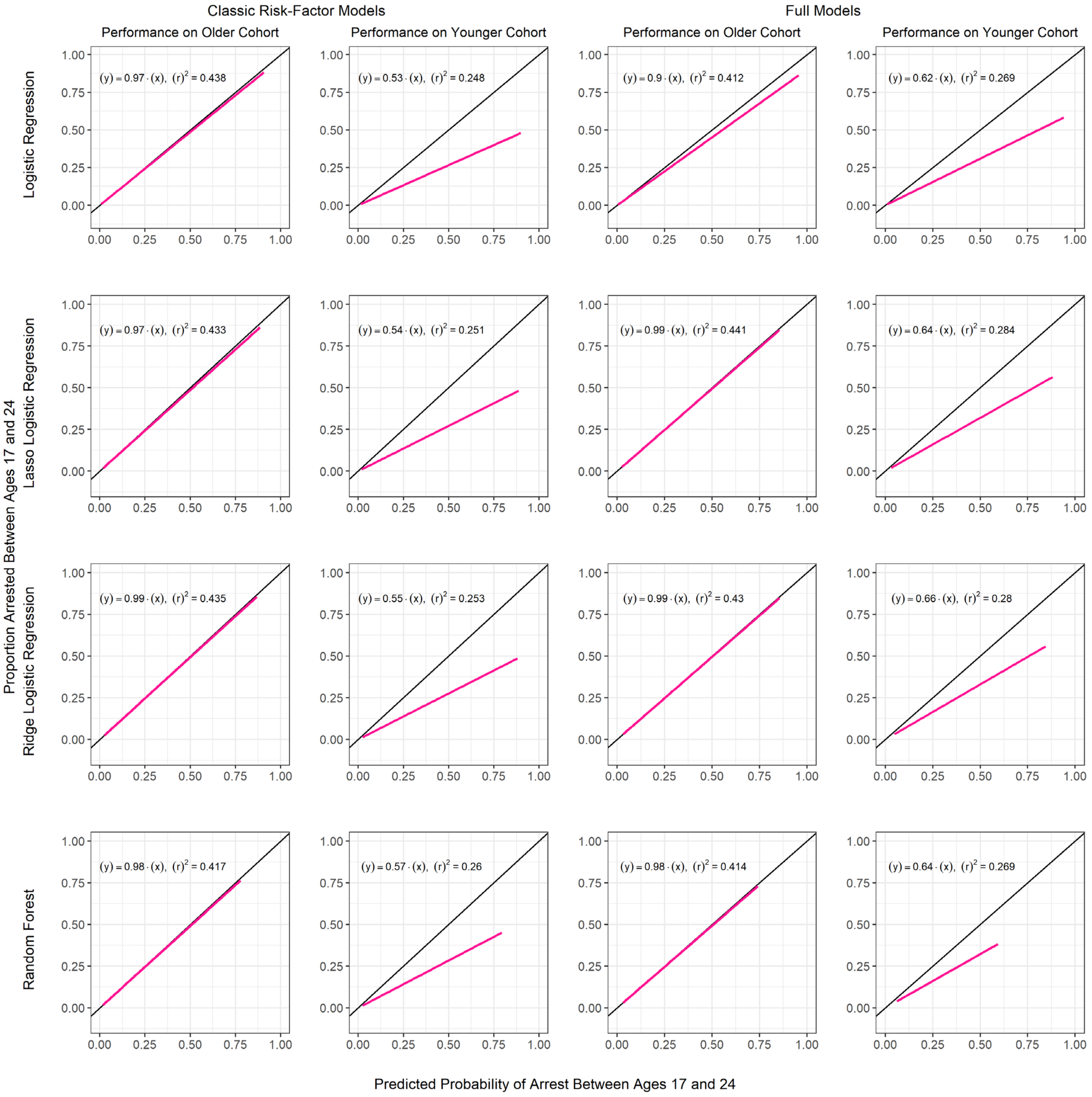

Fig. S1. Calibration plots by model type, feature set, and cohort. The rows show the calibration plots for different model types with the first through fourth rows representing logistic regression, lasso logistic regression, ridge logistic regression, and random forest models, respectively. All models were trained on the older cohort. The first and third columns show the older cohort calibration of these models trained using the classic risk-factor feature set and the full feature set, respectively. These columns show that models trained on the older cohort are well-calibrated to that cohort. The second and fourth columns show the younger cohort calibration of these models using the classic risk-factor feature set and the full feature set, respectively. Regardless of the combination of algorithm or feature set, all models demonstrate cohort bias when applied to the younger cohort.

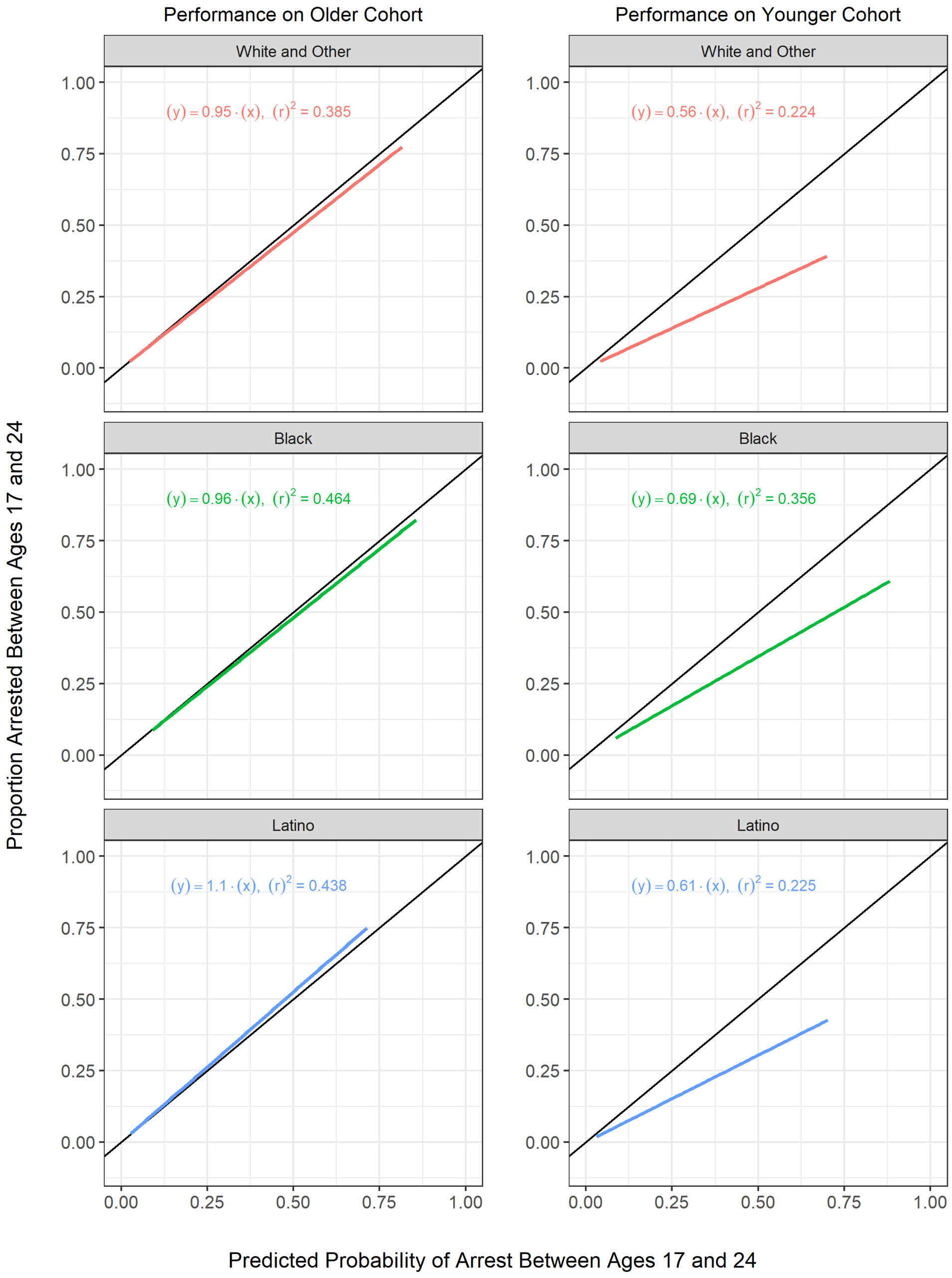

Fig. S2. Calibration plots for full lasso logistic regression model by race. The left column shows the calibration of a full lasso logistic regression model, trained on the older cohort, to that cohort broken out by race/ethnicity. This model is well-calibrated to the older cohort across racial/ethnic groups. The right column shows the calibration of the same model when used to predict for the younger cohort. Here we observe cohort bias for all racial groups, indicating that cohort bias is distinct from racial bias.

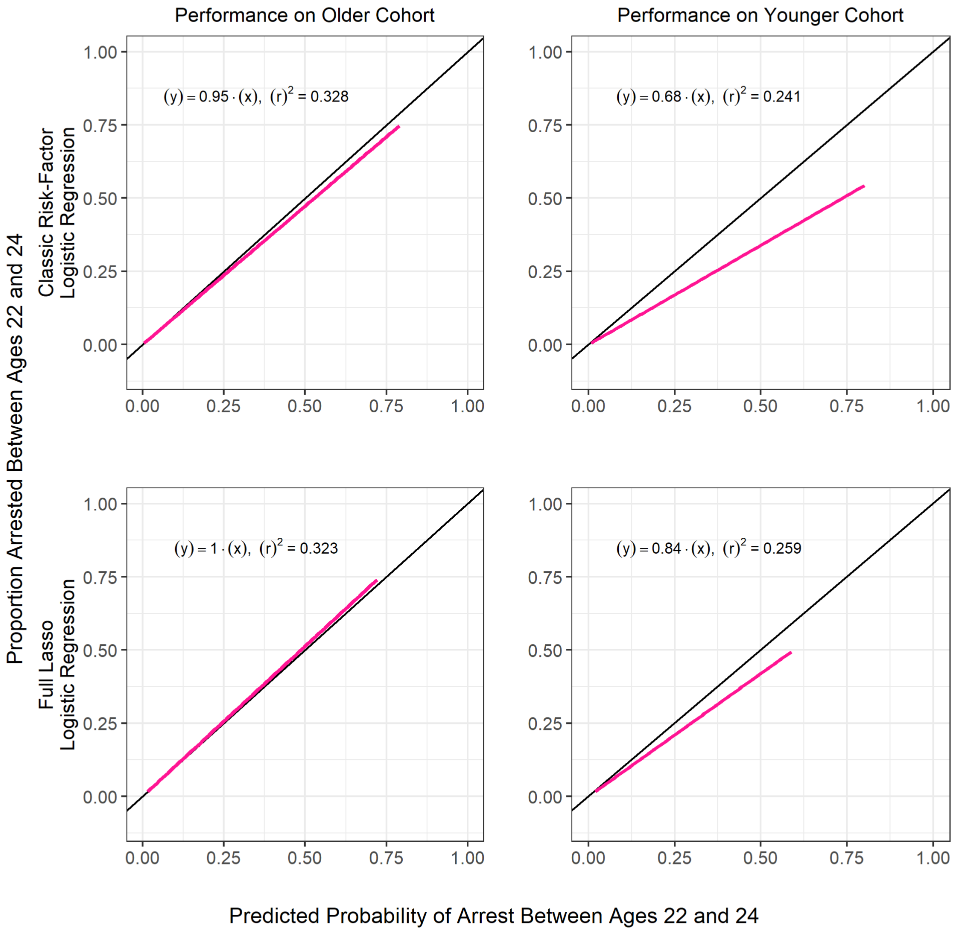

Fig. S3. Calibration plots for models with extended criminal history predictors and a smaller prediction window. The first row shows calibration plots for a logistic regression model trained using the classic risk-factor feature set, and the second row shows calibration plots for a lasso logistic regression model using the full feature set. Both models were trained on the older cohort and include arrest history from age 17-21 as a predictor for arrest between ages 22 and 24. The first column shows the calibration of these two models to the older cohort, and the second column shows the calibration of these two models to the younger cohort. Cohort bias is attenuated somewhat relative to earlier formulations, but it is still present in both models.

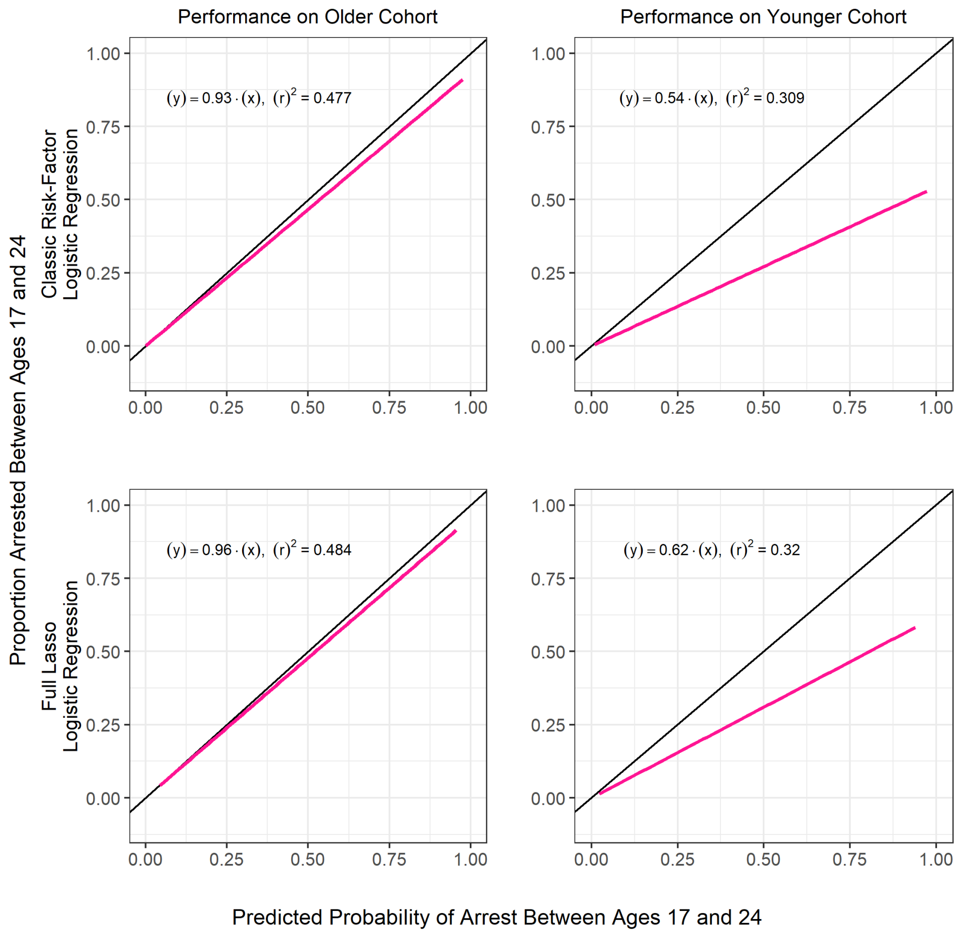

Fig. S4. Calibration plots for models with juvenile criminal history as a predictor. The first row shows calibration plots for a logistic regression model trained using the classic risk-factor feature set, and the second row shows calibration plots for a lasso logistic regression model using the full feature set. Both models were trained on the 9-year-old cohort, the subset of the older cohort for which we have full juvenile arrest records. Arrest history from age 10-16 is used as a predictor for arrest between ages 17 and 24. The first column shows the calibration of these two models to the older cohort, and the second column shows the calibration of these two models to the younger cohort. The addition of juvenile arrest history does not have a substantive impact on the observed cohort bias. The observed calibration slopes for the younger cohort, 0.54 and 0.62, are nearly identical to those observed in the original formulation which did not include juvenile arrest history as a predictor (0.53 and 0.64).

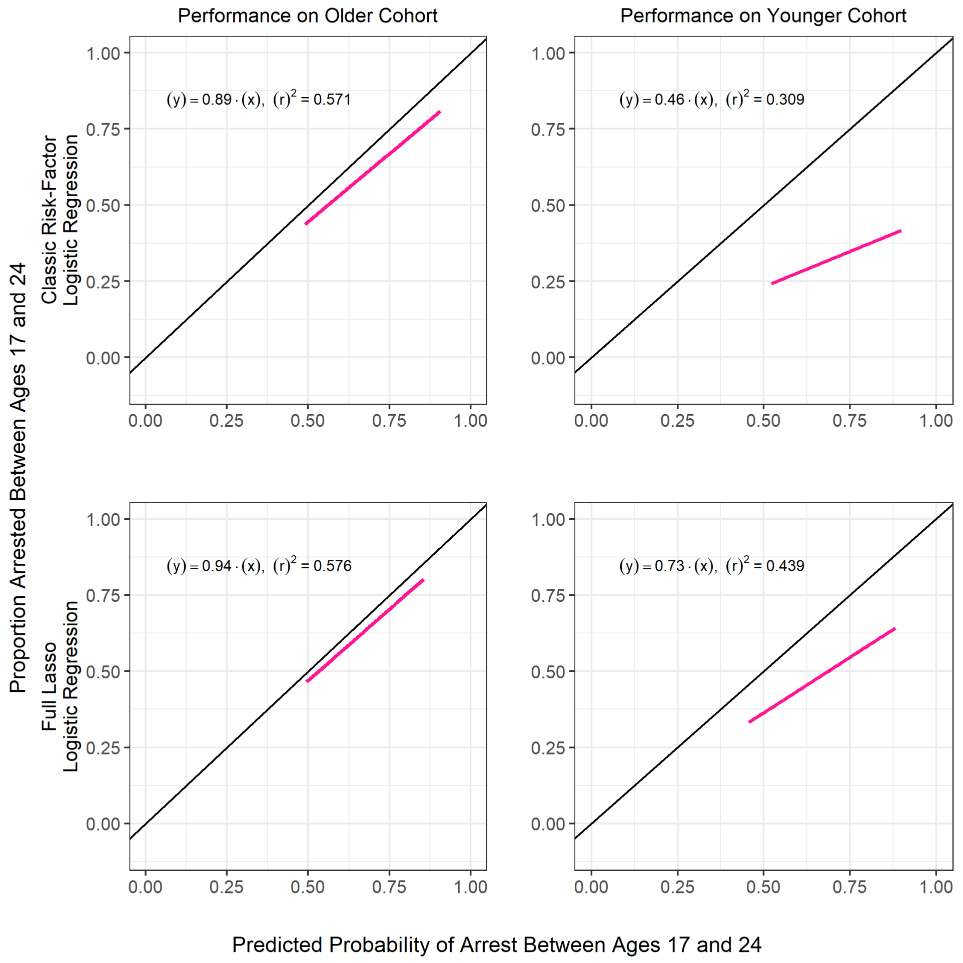

Fig. S5. Calibration plots for individuals with high predicted probability of arrest. These models are trained on the older cohort. The calibration plots include individuals predicted to be in the top quartile for risk of arrest. The top row shows the calibration of the classic risk-factor logistic regression model with performance for the older cohort on the left and performance for the younger cohort on the right. The bottom row shows the performance of full lasso logistic regression model. The deviation of the regression line from the diagonal in the right column indicates that cohort bias is still observed when limiting the calibration analysis to only the highest risk individuals, as defined by predicted arrest probability.

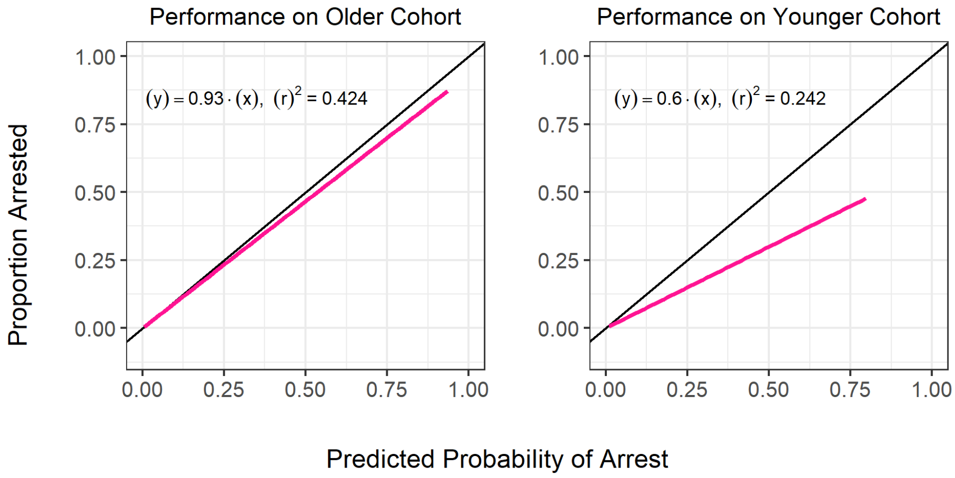

Fig. S6. Calibration plots for a classic risk-factor logistic regression model that includes changing neighborhood characteristics as predictors. These calibration plots show the performance of a logistic regression model trained on the older cohort using the classic risk-factor feature set as well as neighborhood census characteristics collected at ages 9-,13-, and 17-years old. These features do not adequately capture the larger social changes creating cohort bias as their inclusion in the model does not substantially change the degree of cohort bias observed.

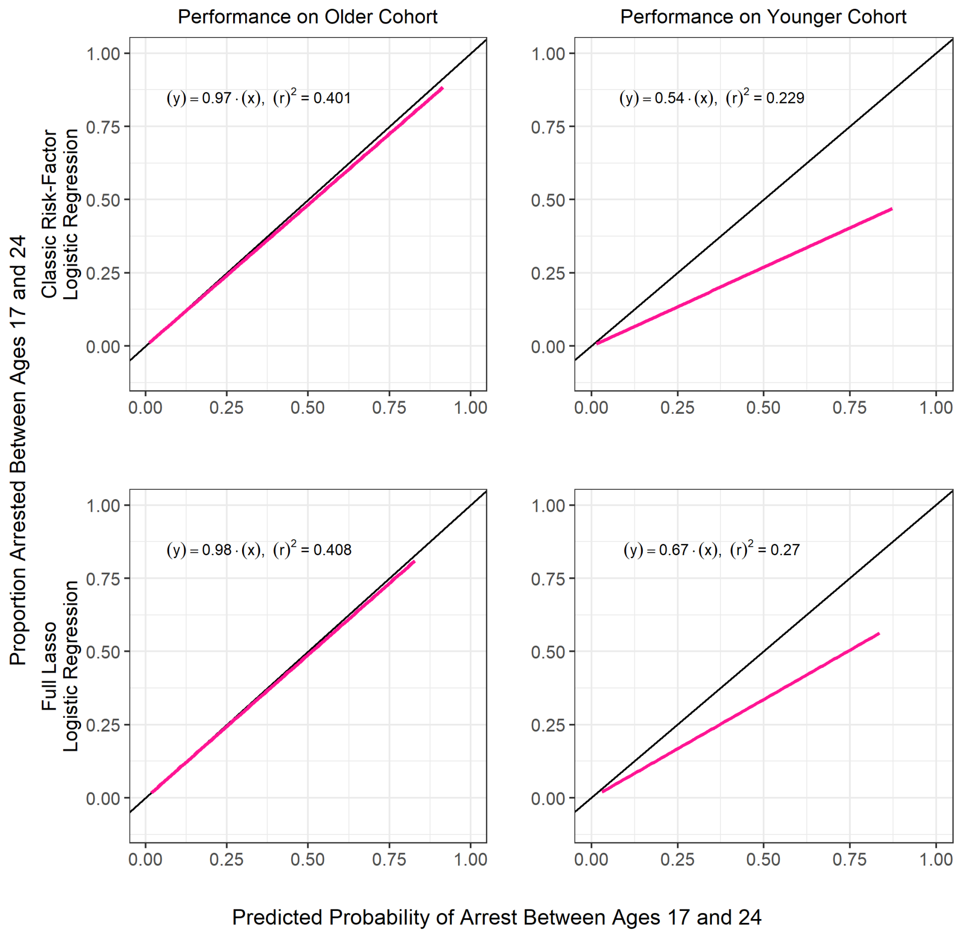

Fig. S7. Calibration plots for models trained to predict the likelihood of non-drug arrests between 17 and 24-years old. The top row shows the calibration of the classic risk-factor logistic regression model with performance for the older cohort on the left and performance for the younger cohort on the right. The bottom row is similar but shows the performance of the full lasso logistic regression model. Both models were trained on the older cohort to predict whether an individual would be arrested on a non-drug charge. While it is possible that the cohort bias observed in earlier formulations was the result of shifting policies, particularly related to highly discretionary charges such as drug offenses, excluding this discretionary arrest category has little impact on the degree of cohort bias, indicating that changes in drug enforcement practices are not the primary driver of cohort bias.
